# Fractional-Order Modeling of Heat and Moisture Transfer in Anisotropic Materials Using a Physics-Informed Neural Network

**DOI:** 10.3390/ma17194753

**Published:** 2024-09-27

**Authors:** Yaroslav Sokolovskyy, Kazimierz Drozd, Tetiana Samotii, Iryna Boretska

**Affiliations:** 1 Department of Computer-Aided Design, Lviv Polytechnic National University, 12 S. Bandery Street, 79013 Lviv, Ukraine; yaroslav.i.sokolovskyi@lpnu.ua; 2 Department of Materials Engineering, Faculty of Mechanical Engineering, Lublin University of Technology, 36 Nadbystrzycka Street, 20-618 Lublin, Poland; 3 Department of Software Engineering, Ukrainian National Forestry University, 103 Gen. Chuprynky Street, 79057 Lviv, Ukraine; t.samotiy@nltu.edu.ua; 4 Department of Computer Science, Ukrainian National Forestry University, 103 Gen. Chuprynky Street, 79057 Lviv, Ukraine; boretska@nltu.edu.ua

**Keywords:** fractal structure, self-organization, heat-and-mass exchange, fractional-differential apparatus, step-by-step learning, fractal neural method

## Abstract

Mathematical models of heat and moisture transfer for anisotropic materials, based on the use of the fractional calculus of integro-differentiation, are considered because such two-factor fractal models have not been proposed in the literature so far. The numerical implementation of mathematical models for determining changes in heat exchange and moisture exchange is based on the adaptation of the fractal neural network method, grounded in the physics of processes. A fractal physics-informed neural network architecture with a decoupled structure is proposed, based on loss functions informed by the physical process under study. Fractional differential formulas are applied to the expressions of non-integer operators, and finite difference schemes are developed for all components of the loss functions. A step-by-step method for network training is proposed. An algorithm for the implementation of the fractal physics-informed neural network is developed. The efficiency of the new method is substantiated by comparing the obtained numerical results with numerical approximation by finite differences and experimental data for particular cases.

## 1. Introduction

The study of the physical processes of interconnected heat-and-mass transfer in fractal anisotropic media is a relevant and interdisciplinary topic that covers physics, mathematics, materials science, geophysics, biomedicine, and other fields. Fractal media have complex geometry, which affects the behavior of physical processes, especially when these processes are in a non-equilibrium state. The construction of effective numerical algorithms for implementing the mathematical models of heat-and-moisture transfer remains an important problem in modern research. Since the case in point is a physical system that has a complex fractal structure and is characterized by such essential properties as memory, self-organization, and spatial non-locality, then, for its mathematical description, it is necessary to use non-traditional methods based on the differential apparatus of fractional calculus.

The mathematical apparatus of fractional integro-differentiation has been known for a long time and is quite developed. However, its use in modeling the physical systems of fractal media has only recently begun. Its application makes it possible to more deeply comprehend the existing known research results and obtain new solutions that take into account the properties of temporal non-locality and spatial self-similarity. Traditional models based on the theory of integer differentiation and Euclidean geometric assumptions are often unable to adequately describe such complex structures with fractal properties. Also, the problems of using fractional differential and integral operators for modeling various processes are associated with a variety of their definitions and the lack of a substantiated physical interpretation.

Various fractional-order differential operators such as Riemann–Liouville, Caputo, and Atangana–Baleanu [1,2,3,4,5,6,7,8,9,10], as well as other fractional calculus concepts like Caputo–Fabrizio, Hilfer, Prabhakar, and Riesz [10,11,12,13,14,15,16], have wide applications in many fields of science and engineering, where it is necessary to model processes with fractal properties, heredity, or anomalous behavior. Fractal mathematical models are used to study physical systems, in particular, viscoelasticity and anomalous diffusion, as well as in the engineering, financial, chemical, biological, medical, and pharmaceutical fields. Fractional models can describe the complex mechanical properties of materials, particularly the behavior of composite materials, polymers, or shape-memory materials. The Caputo fractional derivative [1,4,17,18] has its specific advantages for taking into account the history of the process, hereditary effects, or complex dynamics, especially in cases where the initial conditions are given in the form of classical derivatives.

The Caputo–Fabrizio fractional derivative [9,10,19,20] is a modification of the classical Caputo fractional derivative, which is distinguished by the presence of a kernel with exponential fading, which allows the modeling of systems with limited memory. This derivative has important advantages while modeling systems with memory effects, but without strong singularities at the starting point of the time count. It has found applications in many fields, particularly in those where it is necessary to consider the time dynamics with the gradual fading of hereditary effects. This model is especially suitable for describing heat conduction processes in materials with limited memory [21], such as nanomaterials or composite materials.

The Atangana–Baleanu fractional derivative [4,5,6,7,8] is a modification of fractional derivatives that features a convolution kernel based on the Mittag–Leffler function. It is used to model complex physical, engineering, biological, and economic systems with the properties of heredity and nonlinear dynamics [5,6,22,23]. The advantage of this derivative is that it combines the features of fractional calculus and ensures the smoothness of the models, making them more stable in many applications. The Prabhakar fractional derivative [13,14,15] is one of the new modifications of fractional derivatives, based on the use of the generalized Prabhakar function, which allows the description of processes with non-linear inheritance. This derivative is used to model systems that have complex time delays and hereditary effects that not only fade out over time, but also have complex forms of extinction or resonance.

The Prabhakara derivative is applied in anomalous heat transfer models in cases where materials have complex thermal conductivity properties [11,12,24,25]. This is important for describing processes in composite materials, nanomaterials, or liquids with additional energy states.

The implementation of various mathematical models of heat-and-moisture transfer by analytical methods, such as the Laplace and Fourier transform methods [26,27], etc., are limited in application, and numerical methods, such as the spectral method based on the Laguerre polynomials, finite element method, and finite difference method [28,29,30,31,32], are characterized by high computational complexity and require significant amounts of memory and time. Therefore, there is a need to develop alternative methods. Today, an approach using artificial neural networks (ANNs) is relevant for finding an approximation solution to fractional-order differential equations.

The studies that used ANNs to solve a class of problems described by differential equations began as early as the last century. At the initial stages, researchers used a multilayer perceptron and a cost-valuation function for the numerical calculation of ordinary differential equations [33,34] and partial differential equations with regular boundaries [34]. Later, the method was extended to irregular boundaries [35]. During the next decade, more and more research works appeared with an increased number of parameters and layers of ANNs and with the use of radial-basis neural networks. The next stage in the development of network methods is associated with progress in software, open access to libraries such as the Tensorflow and PyTorch, and the implementation of the automatic differentiation operation [36] in such libraries.

An important and successful approach in the implementation of the numerical algorithms of differential equations is the use of physics-informed neural networks (PINNs) due to the integration of physical laws directly into the architecture of neural networks. The idea of including previously known information about the physical model in the neural network algorithm belongs to Dissanayake and Phan-Thien [37]. The first publications about networks that include information about the physical process as a parameter of the regularization of the cost function appeared in 2017. Two years later, a combined version of these articles was published, in which the authors [38], using the examples of the Burgers, Schrödinger, and Allen–Kahn equations, demonstrated the use of neural networks based on the implementation of physical laws (PINNs) and obtained improved interpolation properties of such networks. However, using PINN in real time is associated with certain difficulties since the network learning is slow and expensive due to the use of optimization algorithm—in particular, gradient descent [39]. There are also challenges with network scaling [40], vanishing gradient [41], solution stuck at local minima, and fine-customizing the PINN learning process, which remains a manual process [38,42]. The authors of [43] tried to improve the effectiveness of the PINN learning process by taking into account the initialization influence factor, and [44] noted the ability of this network structure to effectively learn on a limited data set. This is particularly important for fractal anisotropic media where data may be limited or difficult to access.

The authors of [45] demonstrated that transfer learning can improve the reliability and convergence of PINNs for high-frequency problems, reducing the need for data and training time without increasing the number of network parameters. However, due to the advantages of PINNs, such as consistency with physical constraints, the accuracy of approximation, generalizations, and the ability to learn on sparse data, PINNs have become widely used to solve a diverse class of problems including partial differential equations and integro-differential fractional equations. This applies, in particular, to the study of signal and image processing, associative memory, control and optimization systems, the theory of viscoelasticity, heat-and-moisture transfer solute transport [46], and even the evaluation of wind loads on bridges [47]. Additionally, the PINN model effectively solves direct and inverse problems, as shown by studies [48,49] on the prediction of frictional contact temperature and the estimation of the input data, states, and parameters of dynamic systems based on limited output data.

In further studies, PINNs were adapted to solve stochastic differential equations, and the stability of non-linear systems was analyzed [50]. At the same time, other machine learning methods have also demonstrated success in solving computational mechanics problems. For example, a study using an energy-based approach to partial differential equations combined with a collocation strategy showed that the system’s natural energy can be used as a loss function for solving engineering problems [51]. Additionally, genetic algorithms have been successfully applied to optimize architectures such as deep neural networks and adaptive neuro-fuzzy inference systems. This has improved the accuracy of material property predictions, specifically the fracture energy of polymer/nanoparticle composites [52].

The further development of the studies is the implementation of fractal physics-informed neural networks (fPINNs). fPINNs can be adapted for a wide range of tasks, including modeling heat transfer, fluid flow, wave propagation, and other processes in fractal anisotropic media. Although fPINN is not the only approach for finding solutions to fractional partial differential equations, its relevance in modeling fractal anisotropic media is due to the ability to efficiently and accurately model complex systems, for example, with complex geometry and/or high dimensionality, and the integration of physical laws in the learning process, lower computational costs, and universal implementation. Their application to fractal anisotropic media is the newest direction in the field of machine learning and computational physics. Another variation of a physically determined neural network for finding a solution to fractional differential equations is LaplacefPINN [53]. This neural network method allows a simplification of the loss function and avoids the need to introduce many auxiliary points. In [54], the ANN is improved by using the Hausdorff fractional derivative in the activation function of hidden layers to find a numerical approximation of the diffusion equations with the Hausdorff derivative in the one-dimensional case. In [55], researchers used a neural network combined with the Broyden–Fletcher–Goldfarb–Shanno (BFGS) optimization techniques to solve both linear and non-linear fractal differential equations. The authors of [56] combined the ideas of the Monte Carlo method for calculating integrals and the concept of the method of neural networks that take into account physical laws. This makes it possible to efficiently calculate unbiased estimates for the constraints of fractional integro-differential equations in the loss functional when optimizing neural network parameters.

This study examines the use of a fractal physics-informed neural network (fPINN) to approximate the numerical solution of a system of interconnected the partial differential equations of fractional order that describe heat-and-mass transfer processes in anisotropic media. This is the first study of its kind to provide a comprehensive description of the occurrence of two related phenomena, thermal and physical, in a material with an anisotropic fractal structure.

## 2. Materials and Methods

### 2.1. Equations of Heat-and-Mass Transfer of Fractional Order

To determine the change in temperature and humidity, it is necessary to solve the problem of heat-and-moisture transfer represented by the system of Equations (1)–(5). The mathematical formulation of the two-dimensional process of heat-and-mass transfer in an anisotropic body with a fractal structure includes a system of differential equations [30] with fractional orders of derivatives (1) and (2), with corresponding initial conditions (3) and boundary conditions of the third kind (4) and (5):

(1) cρDτα CT=∑i=12λiDxiβ GLT+ερ0rDτα CU,

(2) Dτα CU=∑i=12aiDxiβ GLU+aiδDxiβ GLT,

(3) $${T|}_{\tau =0}=T_{0}{\left(x\right),\;U|}_{\tau =0}=U_{0}\left(x\right),$$

(4) λiDxγ GLT|xi=0,li+σβiU|xi=0,li−Up=αiT|xi=0,li−tc,

(5) αiDxγ GLU|xi=0,li+αiδDxγ GLT|xi=0,li=βiUp−U|xi=0,li,

where $x=(x_{1},x_{2})$—coordinate vector; $x_{i}$—spatial coordinates, $x_{i}\in \left[0,l_{i}\right]$, i=1,2; τ—time coordinate, $\tau \in \left[0,\tau_{\max}\right]$; T(τ,x)—temperature; U(τ,x)—moisture content; c(T,U)—heat capacity; ρ(U)—density; $\lambda_{i}\left(T,U\right)$ and $a_{i}\left(T,U\right)\;\left(i=1,2\right)$—coefficients of thermal conductivity and moisture conductivity factor; $\sigma =\rho_{0}\left(1-\varepsilon \right)$, where $\rho_{0}$—base density and ε is the phase transition coefficient; *r*—specific heat of vaporization; δ(T,U)—the thermo-gradient coefficient; $t_{c}$—ambient temperature; $U_{p}\left(t_{c},\varphi \right)$—equilibrium moisture content; φ—relative humidity of the environment; $\alpha_{i}\left(t_{c},v\right)$ and $\beta_{i}\left(t_{c},v\right)$—coefficients of heat transfer and moisture exchange, respectively; *v*—the speed of movement of the agent of the environment; Dτα C—the operator of the Caputo derivative of fractional order; Dxβ GL and Dxγ GL—the Grunwald–Letnikov differential operator of non-integer order; α(0<α≤1)—fractional order of the derivative in time; β(1<β≤2) and γ(0<γ≤1)—fractional indicators of derivatives in spatial coordinates.

### 2.2. Approximation of Fractal Operators

There are various approaches to the introduction of fractional integro-differential operations; among them are the approaches of Riemann–Liouville, Weyl, Grunwald–Letnikov, Marchaud, Caputo, etc. Let us consider the operators of the differentiation of non-integer order in terms of Caputo and Grunwald–Letnikov. According to Caputo’s definition of a derivative of fractional order [17], initially, the function U(t) must be differentiated with order n(n>α) and then the result must be integrated with order n−α:

(6) Dtα CU(t)=1Γ(n−α)∫at(t−τ)n−α−1U(n)(τ)dτ,

where Γ(n−α) is a gamma function, α∈ R, n−1<α≤n, n=[α]+1, and [α] is an integer part of α. The time derivative at α→1 coincides with the first-order derivative. If we put n=1 and α=0, then the indicator α will satisfy the condition (0<α≤1) and will characterize the eriditarian process as subdiffusion, in which the movement of particles is slower than during the ordinary (α=1) diffusion. Then Relation (6), using the scheme for the Caputo time derivative described in [18], can be approximated as follows:

(7) Dtα CU(t)≈λ−αΓ(2−α)d0U(tk)−dk−1U(t0)+∑i=1k−1(di−di−1)U(tk−i),

where $d_{i}={\left(i+1\right)}^{1-\alpha }-i^{1-\alpha }$, i=0,1,…,K−1, $t_{k}=\lambda k$, k=0,…,K, $\lambda =\frac{t_{\max}}{K}$. The Grunwald–Letnikov derivative [17] of the function U(t) of order β(β>0) and $x\in \left[a,b\right]$ can be written in the following form:

(8) Dxβ GLU(x)=limh→01hβ∑k=0[x−ah](−1)kβk(x−kh),

where $h=\frac{x-a}{K}$ is the length of the partial interval, determined by the spatial coordinate $x_{k}=ih$, h=0,…,K; [·]—denotes the integer part. For the numerical approximation of the non-integer derivative β with respect to the spatial coordinate *x*, the following relation can be used [17]:

(9) Dxβ GLU(xk)≈1hβ∑i=0kγiβU(xk+1−i),

where $\gamma_{i}=\frac{\Gamma (i-\beta )}{\Gamma (-\beta )\Gamma (i+1)}$. The use of the presented numerical discretization methods of fractional operators allows the transformation of the fractional derivatives represented in Equations (1)–(5) into difference schemes and numerically solving the system of equations using the finite difference method. Additionally, the technique of the numerical approximation of fractional operators will be applied in the fractal physics-informed neural network. These methods will enable a comparative analysis of the numerical approximations obtained using the neural network technique, which includes fractional calculus, and the finite difference method.

### 2.3. The Structure of the Neural Network

The development of a neural network architecture to solve the mathematical model in (1)–(5), which takes into account the fractal structure with memory and self-organization effects, as well as spatial non-locality, is a non-standard and difficult task since the system models two interconnected processes with different behavior. To solve the problem of non-isothermal moisture transfer in capillary-porous media, the advantages of the automatic differentiation method [36] were used to take into account cases when the indicators of the derivatives in spatial and temporal coordinates acquire integer values. This method uses a non-standard approach, transforming the software implementation in such a way that it replaces the range of variables or replaces input values with their derived values and redefines the semantics of the operators to calculate integer-order derivatives according to the chain rule [36] of differential calculus. However, the application of the classical rule of automatic differentiation for fractional calculation is difficult since this version of the rule is expressed in the form of an infinite series. The finite-difference numerical methods of discretization are used for the approximate calculation of the derivatives of fractional order [57]. Let us describe the structure of a fractal physics-informed neural network (fPINN) and how it implements an approach to solving Problems (1)–(5), which takes into account the fractional derivatives of a differential equation. The fPINN includes three main components: a feed-forward neural network, a network that incorporates physical laws, and a feedback mechanism. A feed-forward neural network can be described through a composition of functions that recursively perform both linear and non-linear transformations of the input data in (10) and (11). Let us denote the set of input data as z, and the set of output data as Y(z). Thus, the function Y(z) is approximated using a multilayer feed-forward neural network as follows:

(10) Y(z)≈N(z,μ),

(11) $$N\left(z,\mu \right)=F_{L}\circ F_{L-1}\cdots \circ F_{1}\left(z\right),$$

where z:=(τ,x)—a set of independent variables in space and time, respectively; Y:=(U,T)—a set of the dependent variables of humidity and temperature; N(z,μ)—a neural network working with input features z:=(τ,x); $\mu ={\left\{W_{l},b_{l}\right\}}_{1\leq l\leq L}$—a set of network parameters depending on the weighting factors *W* and displacements *b*, $\mu \in R^{P}$; *P*—the total number of network parameters. The operation ∘ denoting the composition of functions is defined as follows:

(12) F2∘F1(z)=F2X22F1(z),

the functions $F_{l}\left(z\right)$, l=1,2…,L are expressed as follows:

(13) Fl(z)=alX22WlFl−1(z)+bl.

In Expression (13), *l*—the number of the layer of the neural network (1≤l≤L); $W_{l}$—the matrix of weighting factors; $b_{l}$—the vector of displacement; $a_{l}$—the activation non-linear function.

In this case, the neural network must learn to approximate differential equations by defining a set $\mu ={\left\{W_{l},b_{l}\right\}}_{1\leq l\leq L}$. This process is defined through the physics-informed network and the feedback mechanism. Thus, the second functional component of fPINN, based on the obtained output and the given Equations (1)–(5), calculates derivatives to determine the overall network loss $L_{N}$ (14). This is defined by a function consisting of the sum of losses $L_{f}$ for the differential Equations (1) and (2); initial conditions $L_{ic}$ (3); boundary conditions $L_{bc}$ (4) and (5); and, ultimately, some known data ($L_{d}$). Each of these terms is considered with respective weights ($\eta_{d},\eta_{f},\eta_{ic},\eta_{bc}$) to achieve the proper balance:

(14) $$L_{N}=\eta_{d}L_{d}+\eta_{f}L_{f}+\eta_{ic}L_{ic}+\eta_{bc}L_{bc}.$$

In Formula (14), each component is evaluated on the sets of learning points belonging to the set *Z* defined in the region $G=\left[0,\tau_{\max}\right]\times \left[0,l_{i}\right]$, i=1,2. The mean squared error (MSE) method is applied to each component of the loss function to determine the error of each physical state of the system. Accordingly, the losses associated with learning data can be written in the following form:

(15) $$L_{d}=\frac{1}{N_{d}}\sum_{i=1}^{N_{d}}{∥N\left(z_{i}\right)-Y_{i}^{*}∥}^{2},$$

where $Y^{*}$ represents learning data.

The losses $L_{f}$, associated with Equations (1) and (2), are determined as follows:

(16) $$L_{f}=L_{T}+L_{U},$$

where $L_{T}$ and $L_{U}$ are defined as the residuals of Equations (1) and (2), respectively:

(17) LT=1Nf∑j=1NfcρDτα CT|(τj,xj)−∑i=12λiDxiβ GLT|(τj,xji)−ερ0rDτα CU|(τj,xj)2,

(18) LU=1Nf∑j=1NfDτα CU|(τj,xj)−∑i=12aiDxiβ GLU|(τj,xji)+aiδDxiβ GLT|(τj,xj)2.

The losses associated with the initial and boundary conditions, which correspond to Equations (3)–(5), are determined as follows:

(19) $$L_{ic}=L_{T_{ic}}+L_{U_{ic}},$$

where

(20) $$L_{T_{ic}}=\frac{1}{N_{i}}\sum_{j=1}^{N_{i}}{\left(T{|}_{\tau =0}-T_{0}\left(x_{j}\right)\right)}^{2},$$

(21) $$L_{U_{ic}}=\frac{1}{N_{i}}\sum_{j=1}^{N_{i}}{\left(U{|}_{\tau =0}-U_{0}\left(x_{j}\right)\right)}^{2},$$

(22) $$L_{bc}=L_{T_{bc}}+L_{U_{bc}},$$

and $L_{T_{bc}}$ are $L_{U_{bc}}$ defined as the residuals of Equations (4) and (5):

(23) LUbc=1Nb∑j=1NbXYaiDxiγ GLU|xi=0,li−aiδDxiγ GLT|xi=0,li−βiUp−U|xi=0,li2,

(24) LTbc=1Nb∑j=1NbXYλiDxiγ GLT|xi=0,li−σβiU|xi=0,li−Up−αiT|xi=0,li−tc2.

In Expressions (17)–(24), $N_{f}$, $N_{i}$, $N_{b}$ denote sets of learning points $\left(Z=\left\{N_{f},N_{i},N_{b}\right\}\right)$, and, in so doing, $N_{f}$ represents the number of learning points that are located inside region *G*. $N_{i}$ and $N_{b}$ are two sets of learning points located on the boundary of region *G*.

Denote the set of differential operators given in Equations (17), (18), (23), and (24), which define the network losses, as D=Dτα C,Dxiβ GL,Dxiγ GL. When calculating the loss function in (14), it becomes necessary to separate the operators from the set acting on temperature and moisture content into two differentiated groups. This division of operators depends on the values of parameters α, β, and γ. In the case when these parameters have integer values, the operators belong to the first group and, in the case of fractional parameter values, to the second group. The first group includes operators that can be differentiated using the chain rule. The second group includes operators that cannot be subjected to the classical process of differentiation using the chain rule.

In order to exclude the auto-differentiation of operators of the second type, it is necessary to use discrete approximations of fractional differential operators and include them in the loss function [57]. For this, we use the given difference approximations for the fractional operators of Caputo (7) and Grunwald–Letnikov (9), and obtain the approximation of the components of the loss function in (14), which are represented by Expressions (17) and (18). Marking $U\left({\left(x_{1}\right)}_{n},{\left(x_{2}\right)}_{m},\tau_{k}\right)=U_{n,m}^{k}$ and $T\left({\left(x_{1}\right)}_{n},{\left(x_{2}\right)}_{m},\tau_{k}\right)=T_{n,m}^{k}$, $T_{0}$ and $U_{0}$ responding in τ=0, we obtain the following relations:

(25) $$\begin{array}{c} L_{U}=\frac{1}{N_{r}^{\prime }}\times \sum_{k=1}^{K}\sum_{n=1}^{N-1}\sum_{m=1}^{M-1}(\frac{1}{\lambda^{\alpha }\Gamma \left(2-\alpha \right)}\left(d_{0}U_{n,m}^{k}-d_{k-1}U_{n,m}^{0}+\sum_{j=1}^{k-1}\left(d_{j}-d_{j-1}\right)U_{n,m}^{k-j}\right)-\\ \;\;\;\;\;\;\;\;\;\;\;\;\;\;\;\;\;\;\;\;\;\;\;\;\;\;\;\;\;\;\;\;\;\;\;\;\;\;\;-\frac{a_{1}}{h_{1}^{\beta }}\sum_{j=0}^{n}\gamma_{j}^{\beta }U_{n+1-j,m}^{k}-\frac{a_{1}}{h_{2}^{\beta }}\sum_{j=0}^{m}\gamma_{j}^{\beta }U_{n,m+1-j}^{k}-\frac{a_{2}\delta }{h_{1}^{\beta }}\sum_{j=0}^{n}\gamma_{j}^{\beta }T_{n+1-j,m}^{k}-\frac{a_{2}\delta }{h_{2}^{\beta }}\sum_{j=0}^{m}\gamma_{j}^{\beta }T_{n,m+1-j}^{k}{)}^{2}, \end{array}$$

(26) $$\begin{array}{c} L_{T}=\frac{1}{N_{r}^{\prime }}\times \sum_{k=1}^{K}\sum_{n=1}^{N-1}\sum_{m=1}^{M-1}(\frac{c\rho }{\lambda^{\alpha }\Gamma \left(2-\alpha \right)}\left(d_{0}T_{n,m}^{k}-d_{k-1}T_{n,m}^{0}+\sum_{j=1}^{k-1}\left(d_{j}-d_{j-1}\right)T_{n,m}^{k-j}\right)-\\ \;\;\;\;\;\;\;\;\;\;\;\;\;\;\;-\frac{\lambda_{1}}{h_{1}^{\beta }}\sum_{j=0}^{n}\gamma_{j}^{\beta }T_{n+1-j,m}^{k}-\frac{\lambda_{2}}{h_{2}^{\beta }}\sum_{j=0}^{m}\gamma_{j}^{\beta }T_{n,m+1-j}^{k}-\frac{\varepsilon \rho_{0}r}{\lambda^{\alpha }\Gamma \left(2-\alpha \right)}\left(d_{0}U_{n,m}^{k}-d_{k-1}U_{n,m}^{0}+\sum_{j=1}^{k-1}\left(d_{j}-d_{j-1}\right)U_{n,m}^{k-j}\right){)}^{2}, \end{array}$$

(27) $$L_{T_{ic}}=\frac{1}{N_{0}^{\prime }}\sum_{n=0}^{N}\sum_{m=0}^{M}{\left(T_{n,m}^{0}-T_{0}\left({\left(x_{1}\right)}_{n},{\left(x_{2}\right)}_{m}\right)\right)}^{2},\;L_{U_{ic}}=\frac{1}{N_{0}^{\prime }}\sum_{n=0}^{N}\sum_{m=0}^{M}{\left(U_{n,m}^{0}-U_{0}\left({\left(x_{1}\right)}_{n},{\left(x_{2}\right)}_{m}\right)\right)}^{2},$$

(28) $$L_{U_{bc}}=\frac{1}{N_{b}^{\prime }}\times \left(L_{U_{bc1}}+L_{U_{bc2}}+L_{U_{bc3}}+L_{U_{bc4}}\right),\;L_{T_{bc}}=\frac{1}{N_{b}^{\prime }}\times \left(L_{T_{bc1}}+L_{T_{bc2}}+L_{T_{bc3}}+L_{T_{bc4}}\right).$$

The process determining the components of sums in Expression (28) is carried out using the following mathematical dependencies, represented by Formulas (29)–(36):

(29) $$L_{U_{bc1}}=\sum_{k=1}^{K}\sum_{m=0}^{M}{\left(\frac{a_{1}}{h_{1}^{\gamma }}\gamma_{0}^{\gamma }U_{1,m}^{k}+\frac{a_{1}\delta }{h_{1}^{\gamma }}\gamma_{0}^{\gamma }T_{1,m}^{k}-\beta_{1}\left(U_{p}-U_{0,m}^{k}\right)\right)}^{2},$$

(30) $$L_{U_{bc2}}=\sum_{k=1}^{K}\sum_{n=0}^{N}{\left(\frac{a_{2}}{h_{2}^{\gamma }}\gamma_{0}^{\gamma }U_{n,1}^{k}+\frac{a_{2}\delta }{h_{2}^{\gamma }}\gamma_{0}^{\gamma }T_{n,1}^{k}-\beta_{2}\left(U_{p}-U_{n,0}^{k}\right)\right)}^{2},$$

(31) $$L_{U_{bc3}}=\sum_{k=1}^{K}\sum_{m=0}^{M}{\left(\frac{a_{1}}{h_{0}^{\gamma }}\sum_{j=0}^{N}\gamma_{j}^{\gamma }U_{N+1-j,m}^{k}-\frac{a_{1}\delta }{h_{1}^{\gamma }}\sum_{j=0}^{N}\gamma_{j}^{\gamma }T_{N+1-j,m}^{k}-\beta_{1}\left(U_{p}-U_{N,m}^{k}\right)\right)}^{2},$$

(32) $$L_{U_{bc4}}=\sum_{k=1}^{K}\sum_{n=0}^{N}{\left(\frac{a_{2}}{h_{2}^{\gamma }}\sum_{j=0}^{M}\gamma_{j}^{\gamma }U_{n,M+1-j}^{k}-\frac{a_{2}\delta }{h_{2}^{\gamma }}\sum_{j=0}^{M}\gamma_{j}^{\gamma }T_{n,M+1-j}^{k}-\beta_{2}\left(U_{p}-U_{n,M}^{k}\right)\right)}^{2},$$

(33) $$L_{T_{bc1}}=\sum_{k=1}^{K}\sum_{m=0}^{M}{\left(\frac{\lambda_{1}}{h_{1}^{\gamma }}\gamma_{0}^{\gamma }T_{1,m}^{k}-\sigma \beta_{1}\left(U_{0,m}^{k}-U_{p}\right)-\alpha_{1}\left(T_{0,m}^{k}-t_{c}\right)\right)}^{2},$$

(34) $$L_{T_{bc2}}=\sum_{k=1}^{K}\sum_{n=0}^{N}{\left(\frac{\lambda_{2}}{h_{2}^{\gamma }}\gamma_{0}^{\gamma }T_{n,1}^{k}-\sigma \beta_{2}\left(U_{n,0}^{k}-U_{p}\right)-\alpha_{2}\left(T_{n,0}^{k}-t_{c}\right)\right)}^{2},$$

(35) $$L_{T_{bc3}}=\sum_{k=1}^{K}\sum_{m=0}^{M}{\left(\frac{\lambda_{1}}{h_{1}^{\gamma }}\gamma_{j}^{\gamma }T_{N+1-j,m}^{k}-\sigma \beta_{1}\left(U_{N,m}^{k}-U_{p}\right)-\alpha_{1}\left(T_{N,m}^{k}-t_{c}\right)\right)}^{2},$$

(36) $$L_{T_{bc4}}=\sum_{k=1}^{K}\sum_{n=0}^{N}{\left(\frac{\lambda_{2}}{h_{2}^{\gamma }}\gamma_{j}^{\gamma }T_{n,M+1-j}^{k}-\sigma \beta_{2}\left(U_{n,M}^{k}-U_{p}\right)-\alpha_{1}\left(T_{n,M}^{k}-t_{c}\right)\right)}^{2}.$$

In addition, during numerical experiments, the following dependencies between the parameters were assumed:

(37) $$a=a_{1};\;a_{2}=1.1a_{1};\;\lambda =\lambda_{1};\;\lambda_{1}=.82\lambda_{2}.$$

The feedback mechanism is implemented by adjusting the set of network parameters on the training set *Z* according to the overall loss in (14). The process of minimizing the loss is carried out using a gradient optimization algorithm [58]:

(38) $$\mu^{*}=\underset{\mu \in R^{P}}{\arg\min}\;L_{N}\left(Z,\tilde{Y},\mu \right),$$

In order to determine the ranking of optimizer efficiency in the optimization problem (38), we empirically examine in turn such algorithms as Adam, Nesterov-accelerated Adam (NAdam), Root Mean Square Propagation (RMSProp), and stochastic gradient descent (SGD) [58].

In the process of network learning, each epoch means the completion of a full cycle during which the model looks through all the examples on the learning data set once. The model in this process predicts the output, calculates the error, and updates the weights using an optimization algorithm. In the context of applying a fractal physics-informed neural network to achieve accurate results, thousands of learning epochs are usually required.

**Figure 1:**
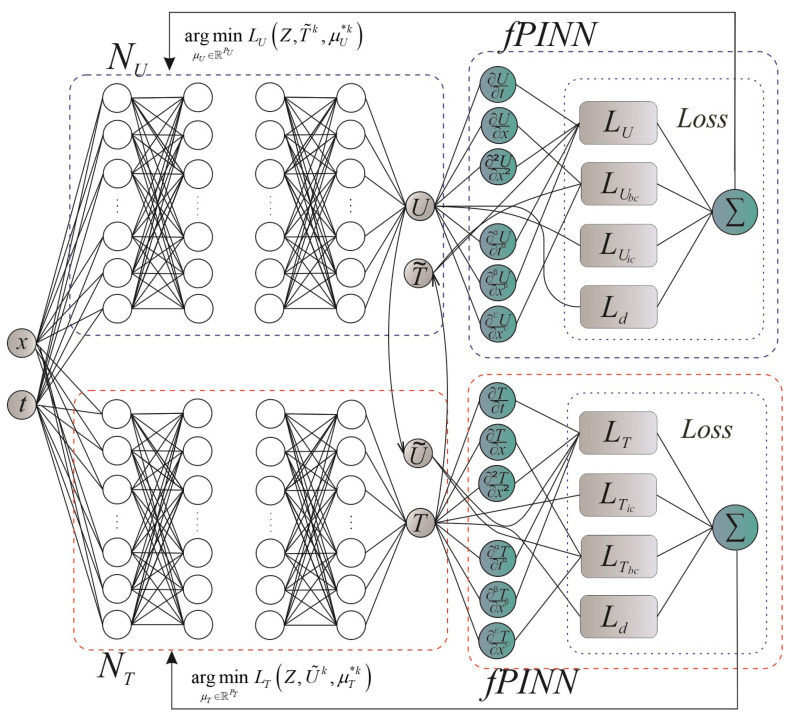
Architecture of neural network model.

However, due to the interconnectedness between the components in the loss function (14), the learning process using gradient optimizers of one neural network to compute two outputs is a difficult task. Therefore, instead of using a single neural network with two outputs, a network architecture is proposed that involves two separate independent feed-forward networks. Each of the networks will have only one output and will be associated with changes in the temperature and humidity potentials in the case when, accordingly, the physical heat flows caused by the interaction of the temperature and humidity gradients are insignificant. This variation of the architectural model influences the number of parameters of independent networks and how different quantities correlate with each other. The process of learning such a model will help reduce vulnerability to errors and failures and speed up computing processes (Figure 1).

Thus, the outputs of separate networks are an approximate solution to Equations (1)–(5):

(39) $$\tilde{U}\left(x,t\right)\approx N_{U}\left(Z,\mu_{U}\right),\;\tilde{T}\left(x,t\right)\approx N_{T}\left(Z,\mu_{T}\right),$$

where $N_{U}$—the neural network for approximating moisture content; $N_{T}$—the neural network for approximating temperature; $\mu_{U}$ and $\mu_{T}$—sets of optimization parameters for two independent networks; *Z*—the total set of learning points in the area $G=\left[0,\tau_{\max}\right]\times \left[0,l_{i}\right]$, i=1,2. The form of the loss functions for $N_{U}$ and $N_{T}$ networks, respectively, in this architecture is as follows:

(40) $$L_{N_{U}}=\eta_{d}L_{d}+\eta_{U}L_{U}+\eta_{U_{ic}}L_{U_{ic}}+\eta_{U_{bc}}L_{U_{bc}},$$

(41) $$L_{N_{T}}=\eta_{d}L_{d}+\eta_{T}L_{T}+\eta_{T_{ic}}L_{T_{ic}}+\eta_{T_{bc}}L_{T_{bc}},$$

where $L_{U}$, $L_{T}$, $L_{U_{ic}}$, $L_{T_{ic}}$, $L_{U_{bc}}$, $L_{T_{bc}}$—the expressions represented by Formulas (25)–(36); $\eta_{d}$, $\eta_{U}$, $\eta_{T}$, $\eta_{U_{ic}}$, $\eta_{T_{ic}}$, $\eta_{U_{bc}}$, $\eta_{T_{ic}}$—weighting parameters for improving optimization algorithms.

The learning process is carried out by minimizing the loss functions of networks $N_{U}$ and $N_{T}$ searching for optimal parameters $\mu_{U}$ and $\mu_{T}$, and is organized as follows. The $N_{U}$ and $N_{T}$ networks learn step by step. The parameters are optimized one by one, first for the $N_{U}$ network, using the fixed parameters of the $N_{T}$ network, and then the actions are performed in the opposite way: parameters are optimized for the $N_{T}$ network with fixed values of the $N_{U}$ network parameters. The process is repeated until the specified level of accuracy is achieved on the learning or validation data set. This process of the optimization problem for the k+1 iteration of learning looks like this:

(42) $$\mu_{U}^{*k+1}=\underset{\mu_{U}\in R^{P_{U}}}{\arg\min}\;L_{U}\left(Z,{\tilde{T}}^{k},\mu_{U}^{*k}\right),$$

(43) $$\mu_{T}^{*k+1}=\underset{\mu_{T}\in R^{P_{T}}}{\arg\min}\;L_{T}\left(Z,{\tilde{U}}^{k},\mu_{T}^{*k}\right),$$

where $\mu_{U}^{*k}$ and $\mu_{T}^{*k}$—the initial parameters of the corresponding networks for the $k^{\mathrm{th}}$ iteration; ${\tilde{T}}^{k}\approx N_{T}\left(Z,\mu_{T}^{*k}\right)$ and ${\tilde{U}}^{k}\approx N_{U}\left(Z,\mu_{T}^{*k}\right)$—an approximation of the output variables at the learning points *Z*.

## 3. Results and Discussion

### 3.1. Algorithmic Aspects of fPINN Implementation

This section demonstrates how the proposed fractal physics-informed neural network can be used for the numerical implementation of the mathematical model in (1)–(5). For the software implementation of this approach, the Python programming language and machine learning tools available in the open source libraries TensorFlow and Keras [59] were used. A description of the typical steps to follow is given to provide a visual representation of the general procedure.

Due to the capabilities of physics-based neural networks to use physics knowledge in learning and to operate with general non-linear equations with partial and fractional derivatives, they are able to effectively solve problems with a limited amount of data (or a set of noisy data) or to recover solutions without a set of learning data. Taking into account this property, when conducting the studies, small amounts of experimental data from [60,61] were used or the constructed models were used as surrogates, i.e., when the unsupervised learning method was applied solely based on physical equations and initial and boundary conditions.

To compare the results obtained by the neural network method and the finite difference method, learning points were generated corresponding to the grid points of the finite difference method. In other cases, a uniformly distributed arrangement of points in the inner region and on the boundary of the region is chosen. The learning points for the test set were chosen according to the experimental data described in [60,61]. Before starting network learning, network weights were initialized using Xavier’s pseudo-random initialization method [62].

A model consisting of two separate fully connected neural networks, which are united by a common input, has been created. It is important to note that both networks have the same configuration. Each of these networks includes 8 hidden layers, where each layer contains 40 nodes. As a result, the total number of parameters in each network is 11,641. In the input layer, there are input parameters (variables’ spatial and temporal coordinates) that provide input data to the network for processing. On the other hand, there is one output in the output layer of each independent network, which is used to approximate and predict the values of moisture or temperature changes.

A hyperbolic tangent has been selected to describe the activation function that was applied to all hidden layers. This function is smooth and continuous, with non-zero derivative and centered with respect to zero. These characteristics of the activation function help avoid “vanishing gradients” problems when the neural networks are learning.

To optimize the loss function, the following algorithms were used: Adam, Adam with Nesterov momentum, root mean square distribution, and stochastic gradient descent. The studies were carried out on a computer station.

The training time for this network configuration can vary significantly depending on a range of factors, such as the volume of training data and training parameters, including the number of epochs and learning rate. Fractal physics-informed neural networks also require additional computational resources for calculating derivatives with respect to time and spatial coordinates, as well as for the numerical implementation of fractional differential equations during training. To measure the training time, we used the time library in Python and built-in TensorFlow tools to monitor the training process.

Let us present the algorithmic aspects of the implementation of the fractal neural model with sequential learning in the context of implementing the heat-and-mass transfer problem in (1)–(5). The following paragraphs present code snippets from our work that illustrate key stages such as model initialization, the calculation of residual errors with the consideration of fractal properties, the definition of the loss function, and the determination of the number of training iterations.

Step 1. We determine the points of the learning data sets $N_{f}$, $N_{i}$, $N_{b}$ for Equations (1) and (2), boundary (4) and (5) and initial conditions (3), and learning data $N_{d}$;

Step 2. According to Figure 1, we describe the function for creating and initializing the feed-forward network model (Listing 1) by specifying the number of hidden layers, the number of neurons in each layer, the activation function for each layer, and the method for initializing the weights for the initial determination of the parameters $\mu_{U}$ and $\mu_{T}$;

**Listing 1.** Initialization of feed-forward model.

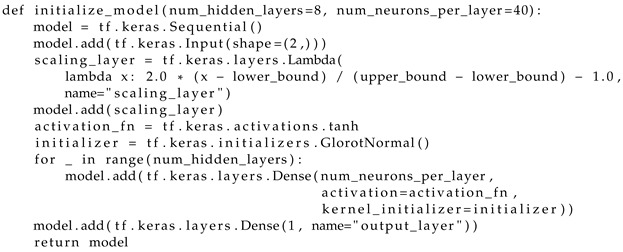



Step 3. Create two separate instances of the neural network models $N_{U}$ and $N_{T}$ using the initialization function from the previous step;

Step 4. Create a function that describes the residuals of the fractional differential equations in (25) and (26), presented as difference schemes, using the capabilities of Tensor-Flow (Listing 2);

**Listing 2.** Computation of residuals.

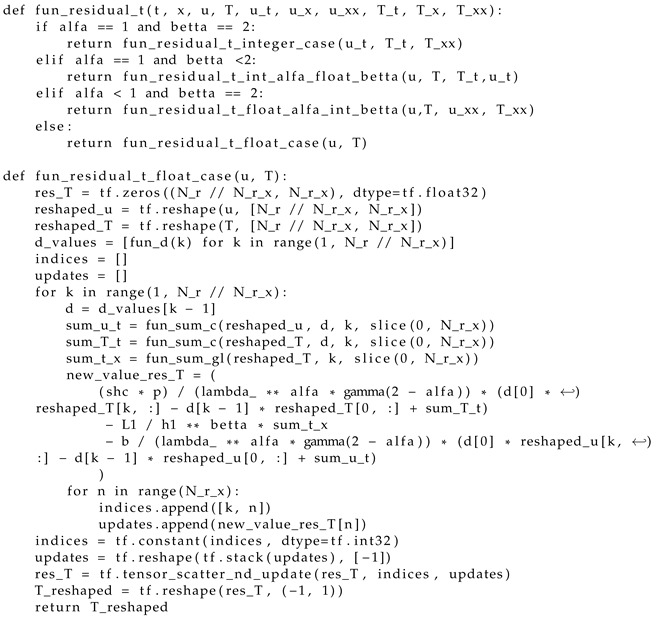



Step 5. Similar to the previous step, describe the residuals of the boundary conditions (28) and the initial conditions (27) using TensorFlow functionalities;

Step 6. We formulate the loss functions in (40) and (41), which are defined as the weighted root mean square error of the remainders of the fractional differential equations in (25) and (26), boundary (28) and initial (27) conditions, and the difference between the network output and test data (15) (Listing 3);

**Listing 3.** Loss function determination.

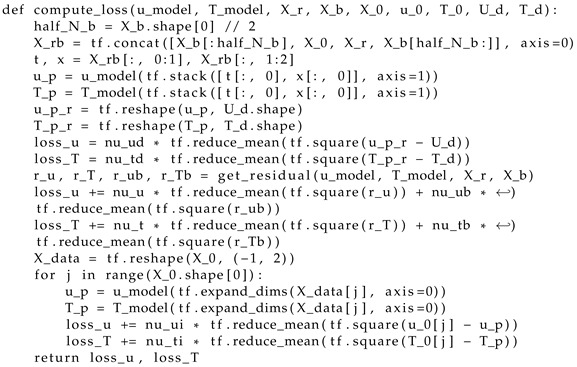



Step 7. For the models determined in step 3, taking into account step 6, we determine the learning procedure using (41) and (42);

Step 8. We set optimization hyper-parameters, such as optimization function and learning rate. We determine the weighting coefficients from Formulas (40) and (41);

Step 9. Determine the number of learning iterations and/or set the error value to stop the iterative process (Listing 4);

**Listing 4.** Finding the number of learning iterations.

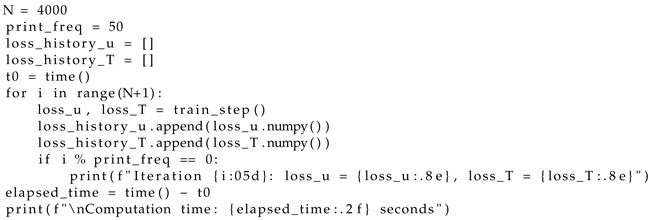



Step 10. Conduct the step-by-step training procedure in (41) and (42) of the networks $N_{U}$ and $N_{T}$ with random initialization. During the training process, the values of the loss functions for each model and the time required for training are calculated and recorded. Use callbacks to monitor and adjust the training process;

Step 11. Use the trained models from the previous step to obtain model predictions and evaluate the solutions of the fractional differential equations on the test data;

Step 12. Visualization of the obtained results.

### 3.2. Research on Temperature and Moisture in Anisotropic Materials

Using the developed neural network model and the given algorithm, a set of experiments was carried out to study the dynamics of spatial heat-and-mass transfer in capillary-porous materials during hydrothermal treatment. The material used was wood with different values of density and various thicknesses (5 mm, 15 mm, 20 mm, 25 mm), depending on the particular test and experimental data availability. The initial parameters of the model were set to the following values: initial sample temperature $T_{0}=20\;{}^{\circ}C$, ambient temperature $T_{0}=70\;{}^{\circ}C$, initial moisture content $U_{0}=0.4\;kg\;{kg}^{-1}$, relative air humidity φ=60%, drying agent speed $v=\;2\;m\;s^{-1}$, material density $\rho =500\;kg\;m^{-3}$. The rest of the parameters and the required empirical relationships were obtained on the basis of experimental data [30,32,60,61,63,64]. In the absence of other instructions, the following values of fractional parameters of the model are set: α=0.82, β=1.92, γ=0.9.

To evaluate the accuracy of the created model, a test experiment was carried out for a one-dimensional fractal problem of heat-and-mass transfer, which was derived from the considered two-dimensional mathematical model. A comparison of the output results of the network for moisture and temperature with known data from other researchers [30,32,60,61,64] was conducted.

**Figure 2:**
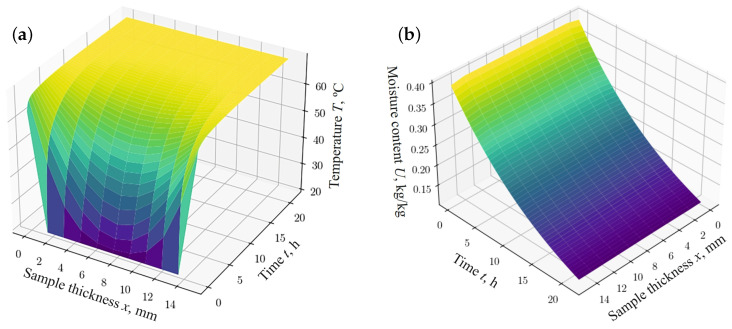
The results obtained using the network model for different thickness of the sample: (**a**) changes in temperature fields; (**b**) changes in moisture content fields.

**Figure 3:**
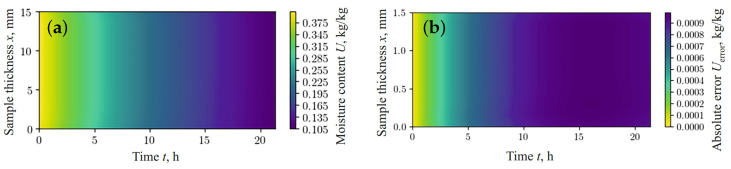
Moisture content obtained using FDM (**a**) and absolute error in predicted moisture content between fPINN and FDM (**b**).

The dependencies shown in Figure 2 and Figure 3 describe the process of thermodiffusion (non-isothermal moisture transfer), considering the fractal characteristics of the material. Considering fractal properties is necessary, as they reflect the complex structure of the material, which directly affects heat transfer and mass transfer processes. For example, fractal structures can either slow down or accelerate diffusion processes, depending on the degree of porosity or the inhomogeneity of the material.

**Figure 4:**
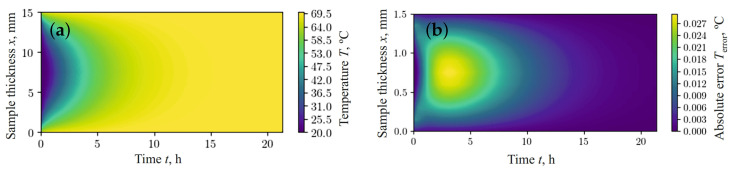
Temperature obtained using FDM (**a**) and the absolute error of predicted temperature between fPINN and FDM (**b**).

Figure 3b and Figure 4b show the absolute error between the values simulated using the finite difference method (FDM) (Figure 3a and Figure 4a) and the results obtained using the fractal network model (Figure 2a,b), respectively. According to the data obtained, the relative error in predicting moisture content and temperature, using the fractal network fPINN and FDM, remains within the limits of satisfactory accuracy for the case under consideration.

**Figure 5:**
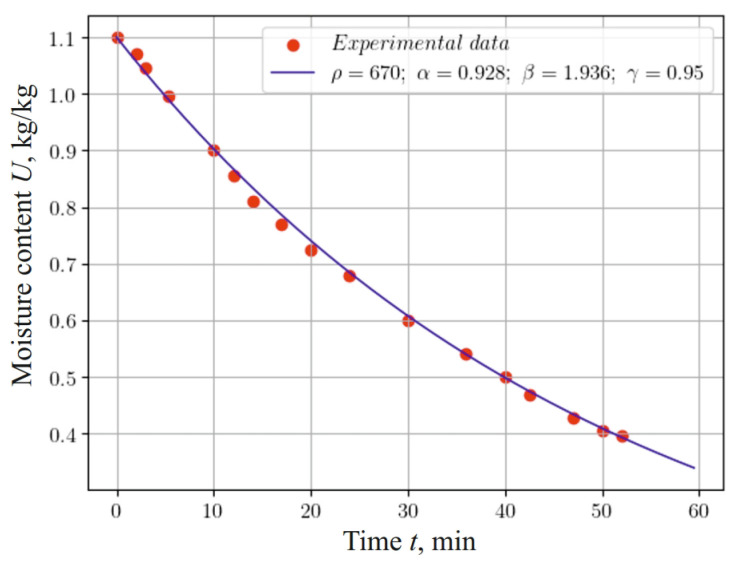
Change in moisture content at the center of a 5 mm thick sample, taking into account the fractal structure of the material and experimental data.

When analyzing the graphs in Figure 5, which reflect changes in moisture content during 60 min of the process of the hydrothermal treatment of a material sample at an ambient temperature of 120 °C, considering the fractal structure of the material (α=0.928, β=1.936, γ=0.95), we can conclude that taking into account the eridity and internal self-organization of the material by using fractional indicators in the mathematical model and the use of a neural network approach allow for fairly accurate modeling of the heat-and-mass transfer process. This testifies to the adequacy of the model and the method of numerical implementation.

**Figure 6:**
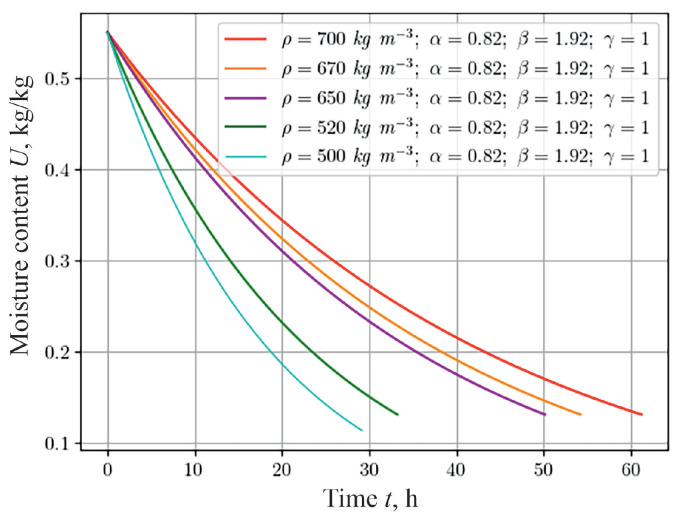
Change in moisture content at the center of the sample, depending on the time for the various material densities.

The temperature dynamics and moisture content of capillary-porous materials during hydrothermal treatment depend on the density of the material, which indicates the degree of porosity. To analyze this dependence for materials with a fractal structure that were 20 mm thick with different densities (namely, 500 $kg\;m^{-3}$, 520 $kg\;m^{-3}$, 650 $kg\;m^{-3}$, 670 $kg\;m^{-3}$, 700 $kg\;m^{-3}$), a numerical study was conducted, the results of which are shown in Figure 6 for the moisture content indicator. The result obtained, in particular, shows that moisture is removed faster from material with lower density. This is due to the heterogeneous structure of the material and the ability to self-organize, causing materials with less density restore their state more slowly.

We investigate the influence of environmental parameters during hydrothermal treatment on changes in temperature and moisture content in a sample of capillary-porous material with a fractal structure.

**Figure 7:**
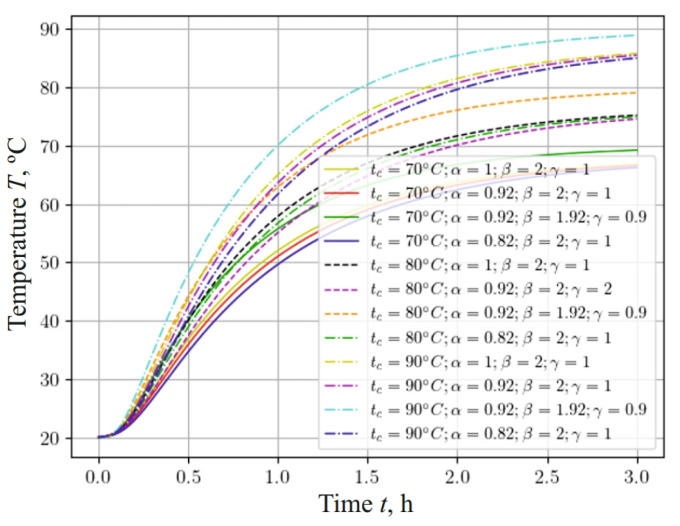
Change in temperature content at the center of the sample, depending on the time for different temperatures of drying agent and fractal parameters of the material.

Figure 7 represents a graph showing how the temperature of a sample with a base density of 460 $kg\;m^{-3}$ changes, taking into account the fractal structure of the material and the different temperatures of the drying agent: 70 °C, 80 °C, and 90 °C. Analyzing this graph, it can be noted that, as the temperature of the agent increases, the intensity of the sample heating increases. It is important to note that the influence of the fractal structure of the material—namely, the fractional-differential parameters α and β of the model—on the temperature dynamics becomes more significant when increasing the temperature conditions of the process.

The temperature in the center of the sample increased faster when using the fractional parameter in the numerical experiment compared to using the integer parameter. The temperature indicator reached about 90 °C much faster when taking into account the fractional parameter β, which contributes to the faster removal of moisture from the porous material. The analysis of the results of the numerical experiment, taking into account the fractal and integer parameters, showed that, with a decrease in the value of the parameter β, the temperature of the capillary-porous material rose more quickly to the temperature of the drying agent. However, a consideration of the fractional exponent α demonstrates a slight slowdown in the heating of the sample, which, in turn, slows down the process of removing moisture from the material.

**Figure 8:**
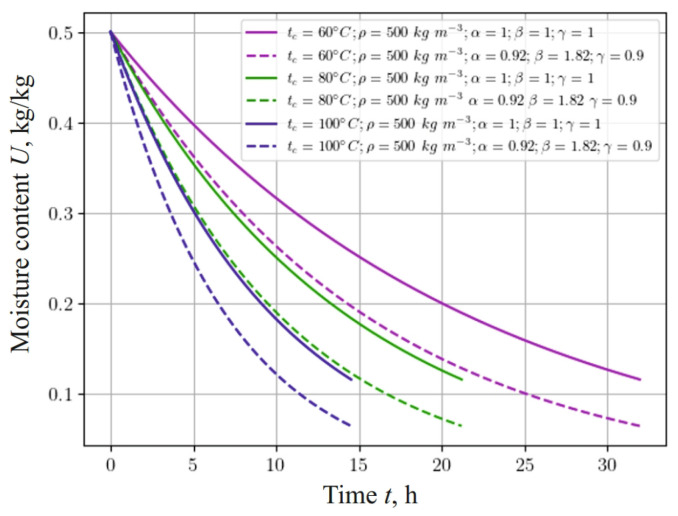
Change in moisture content at the center of 20 mm thick sample, depending on the time for different ambient temperatures and fractal indicators of the material.

Figure 8 represents the dependence, which shows changes in the moisture content of a sample with a density of 500 $kg\;m^{-3}$, taking into account the fractal structure of the material and different values of the temperature of the drying agent: 60 °C, 80 °C, and 100 °C. It should be noted that moisture in the material is released faster with a decrease in the fractional-differential indicators of the model in the process of heat-and-mass transfer in anisotropic media. In particular, taking into account the fractal structure of the capillary-porous material, the process of moisture release is accelerated, that is, the material dries faster, and the moisture content asymptotically approaches the equilibrium value.

**Figure 9:**
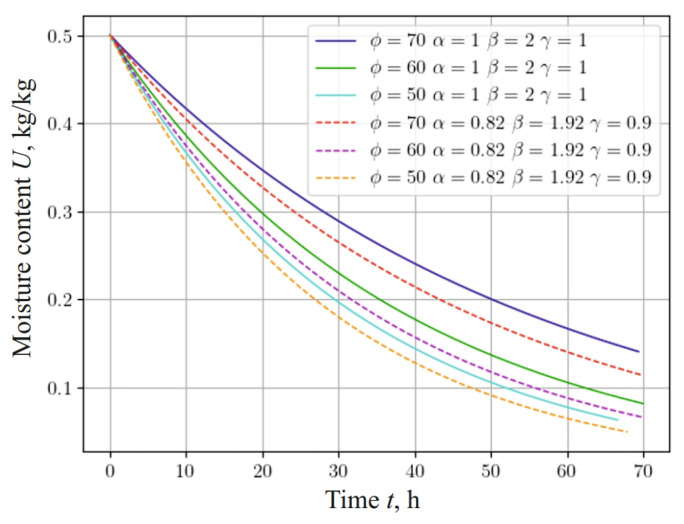
Change in moisture content at the center of 25 mm thick sample, depending on the time for various relative humidities of the environment and fractal indicators of the material.

A study was also conducted on the influence of the relative humidity of the drying agent on the change in the moisture content of the material. The hydrothermal process was simulated for a material with a density of 500 $kg\;m^{-3}$ at an ambient temperature of 70 °C and a drying agent speed of $v=2\;m\;s^{-1}$. During the study, the relative humidity was 50%, 60%, and 70%. The obtained results are presented in Figure 9.

The results of the study took into account the fractal structure of the capillary-porous material. It is obvious that, with a decrease in the relative humidity of the environment, the process of moisture release accelerates, and the influence of the fractal structure of the material on the change in moisture content becomes less significant. This numerical experiment confirms the significant influence of the fractal structure of the material on the dynamics of the moisture content.

**Figure 10:**
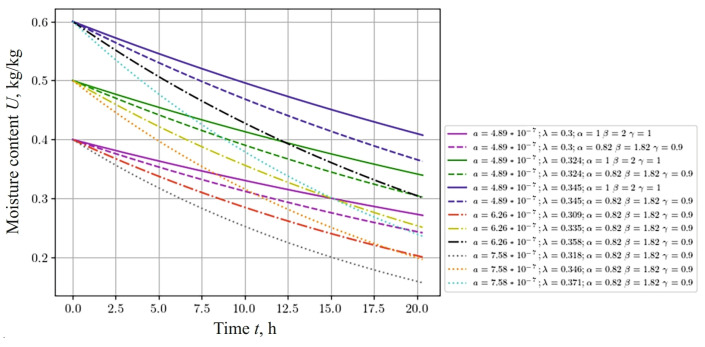
Change in moisture content at the center of the sample, depending on the time for different anisotropic coefficients of moisture, thermal conductivities, and fractal indicators of the material.

Let us investigate how the change in moisture content depends on the anisotropy of the thermophysical properties of the capillary-porous material. In this case, we consider the coefficients that are used in the mathematical model in (1)–(5). These coefficients are anisotropic, that is, their values change, depending on moisture content and temperature. Among them, the following coefficients can be distinguished: thermal conductivity $\lambda_{1}$, $\lambda_{2}$ and moisture conductivity $a_{1}$, $a_{2}$, heat exchange and moisture exchange, equilibrium moisture content and density, and thermogradient coefficient. The ratio for their calculation is given in the source [60,61,63]. Analyzing the graphs presented in Figure 10, the following conclusions can be drawn regarding the change in moisture content. With an increase in the values of the anisotropic coefficients of moisture conductivity and thermal conductivity, a decrease in the moisture content in the material is observed. In addition, it should be noted that the fractal structure of the material influences the dynamics of moisture content. With an increase in the values of the studied coefficients, the influence of the fractal structure of the material on moisture content tends to decrease.

Comparing Figure 8, Figure 9 and Figure 10, one can see a significant influence of the sample thickness on the moisture transfer phenomenon, which is also related to heat convection.

### 3.3. Evaluation of fPINN Architecture Efficiency and the Use of Optimizers

**Figure 11:**
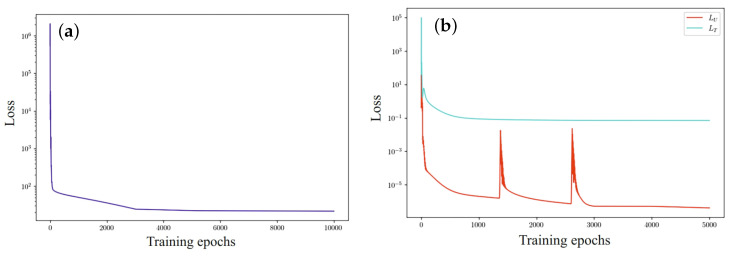
Evolution of loss functions: (**a**) one loss function for two architecturally separated networks; (**b**) two separated networks and disconnected loss functions.

Experimental results evaluating the effectiveness of the selected network architecture are shown in Figure 11. The left part of the figure shows the evolution of one loss function for two architecturally separated networks. Figure 11b shows the results of step-by-step learning of two separated networks and disconnected loss functions.

In the context of multi-objective optimization, where gradients are unbalanced, weighting factors are used to improve the optimization algorithm. These weighting factors are determined during network learning by analyzing the convergence of the optimization process in order to reduce the dynamics of the flow of gradient values.

**Figure 12:**
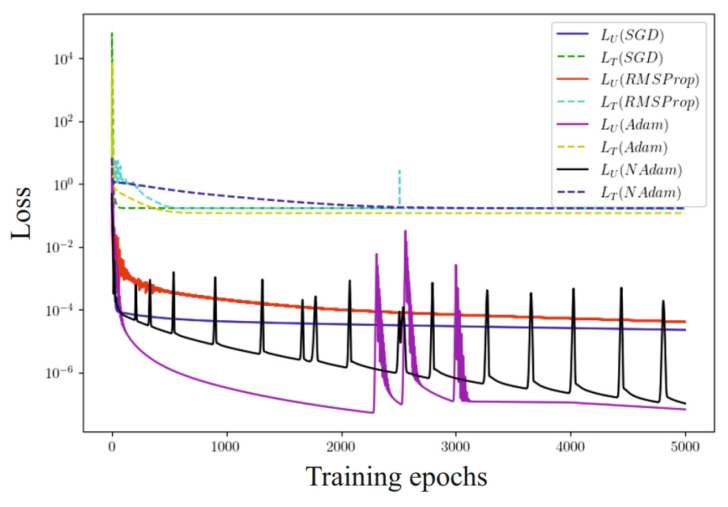
Evolution of the loss function with the use of SGD, RMSProp, Adam, and NAdam optimizers.

During the course of the studies, an analysis of the influence of of several optimization methods was carried out (Figure 12), including methods for estimating the adaptive moment (Adam), the Nesterov-adaptive moment (NAdam), stochastic gradient descent (SGD), and the application of the root mean square of of the gradient (RMSProp) for the output task.

It was found that the use of the Adam optimizer ensures the speed of achieving results and the highest accuracy in all test tasks. The results shown by the stochastic gradient descent (SGD) and RMSProp algorithms are approximately identical. Since the initial sample is not balanced, this in turn negatively affects the performance of these methods. The stability of the learning rate parameter for the SGD method and the regulation of the learning step by the accumulated rate of gradient change at the previous stages for the RMSProp method ensured reaching the local optimum. The adaptive moment method uses not only the mean value (first moment) of the gradients, but also the mean squared value of the gradients (second moment) to adapt the learning rate of the parameters. Also, this method updates the learning rate for each network weight individually and has a non-zero initial calibration. The adaptive moment with impulse unexpectedly shows lower accuracy with longer computation time compared to the traditional Adam method. This means that the value of the received impulse must be optimized. From this, it can be concluded that Adam’s method can find an efficient solution with fewer iterations than the other considered optimization methods. The obtained results partially support the assumption that more universal optimizers shall not perform worse than those they can approximate. However, since the setting-up effort can be disproportionately large, then the use of the above algorithms is not always practically expedient, especially in the context of comparing optimizers such as Adam and NAdam.

**Figure 13:**
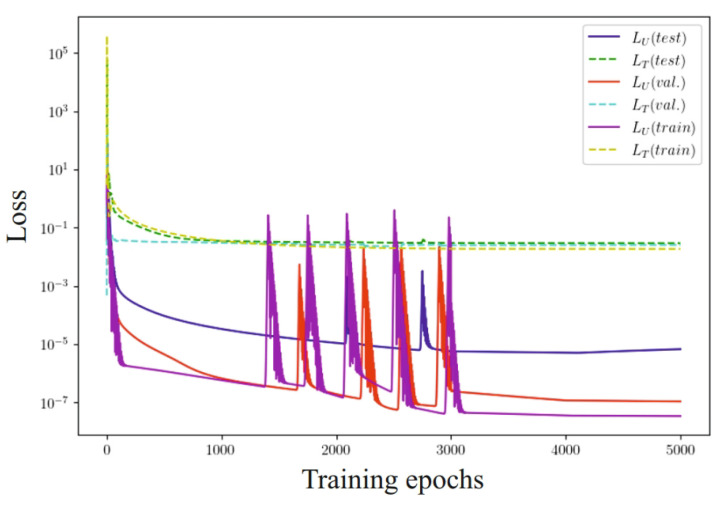
Evolution of loss functions for learning, validation, and test data sets.

To effectively evaluate the model, the data were divided into three main sets: the training set, the validation set, and the test set. The model was trained on the training set, and the loss function was used to optimize its parameters. The validation set was used for hyperparameter tuning and monitoring for overfitting. The loss function on this set helped adjust the model and prevent overfitting. After the training and validation phases, the model was assessed on the test set. The loss function at this stage provided a final evaluation of the model’s generalization ability. Analyzing the loss functions, particularly those presented in Figure 13 and related to the stages of training, validation, and testing, helped in selecting the optimal number of epochs (≈4000) to avoid overfitting. 

### 3.4. Evaluation of the Impact of Data Noise

Let us investigate the impact of noisy data on the system. Let us add the Gaussian white noise with zero mean and root-mean-square deviation $\sigma_{\mathrm{noise}}$ to the learning data set of a one-dimensional problem and obtain $Y_{\mathrm{noise}}^{*}=Y^{*}+\varepsilon_{\mathrm{noise}}$, where $\varepsilon_{\mathrm{noise}}=N\left(0,\sigma^{2}\right)$. Accordingly, Formula (15) will take the form $L_{d_{\mathrm{noise}}}=\frac{1}{N_{d}}\sum_{i=1}^{N_{d}}∥N\left(z^{i}\right)-Y_{i\;\mathrm{noise}}^{*}∥$, while the other parameters of the model will not change.

We used L2-regularization [65] to reduce the impact of noise and to smooth the data. For this, we added a penalty term to the loss functions in (40) and (41) in the form of the sum of the squared values of all model parameters (weights and shifts) multiplied by the constant λ. Adding this penalty term forces the model to seek a trade-off between prediction accuracy and parameter simplicity, which can improve its ability to generalize new data. To determine the percentage of noise in the data, we used the following approach: $k=\frac{\sigma_{\mathrm{noise}}^{2}-\sigma^{2}}{\sigma^{2}}$, where σ is the mean square deviation of the data without noise, which determines the amount of value spread in “clean” data. To evaluate the obtained results, we used the relative error:

(44) $$\delta =\frac{1}{N_{d}}\sum_{i=1}^{N_{d}}\frac{\left|N\left(z^{i}\right)-Y_{i}^{*}\right|}{Y_{i}^{*}}.$$

**Figure 14:**
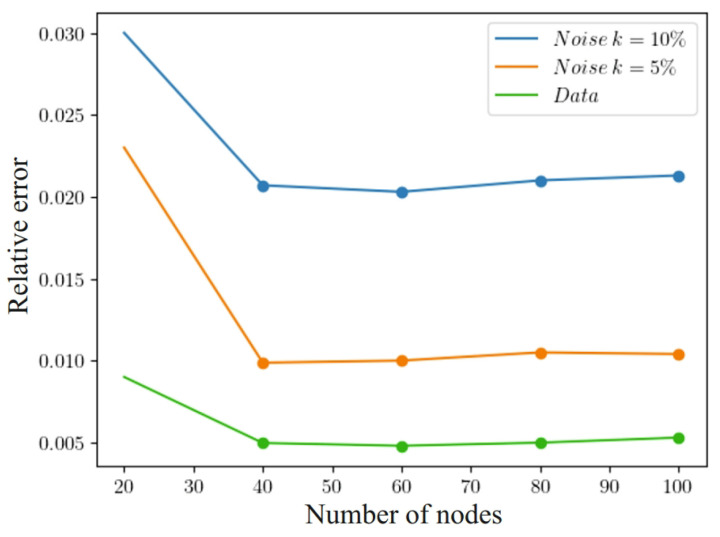
Influence of noisy data on solution accuracy.

Figure 14 shows how the impact of noisy data affects the accuracy of the system solution in (1)–(5). A noise level of 5% allows it to reach almost 2.3% relative error. In addition, to accurately recover the concentration fields with an accuracy of about 1% at a noise level of 5%, it is necessary to have at least 40 learning points in each hidden layer. The model exhibits the best regenerative qualities when there are 60 nodes in each hidden layer. An increase in the number of nodes in the model leads to a deterioration of its ability to generalize, which is associated with a limited amount of learning data.

## 4. Conclusions

In this article, two-dimensional mathematical models of heat and moisture transfer, taking into account the anisotropy of the material’s thermophysical properties, based on the use of the fractal calculus of integro-differentiation is presented. To describe these models, the fractional orders of derivatives with respect to time and space coordinates, defined in terms of the Caputo and Grunwald–Letnikov derivatives, are used. A neural network method for implementing the mathematical model has been developed based on the architecture of a fractal physics-informed neural network with a decoupled structure and step-by-step training. This method allowed for the investigation of trends in the changes of the material’s moisture and temperature, considering memory effects, spatial non-locality, and self-organization, as well as overcoming pronounced imbalances in the training process. Loss functions containing information about the physics of processes are introduced, and different schemes for loss functions are constructed based on formulas for the numerical approximation of the fractional operators of the model.

A comparison is made between the numerical results obtained using the fractal network and the results obtained using numerical finite difference methods. This comparison demonstrates the acceptable accuracy of the developed method. The gradient optimizer was selected based on the analysis of numerical experiments. An algorithm for the implementation of the fractal neural model has been developed. Comparing the obtained results with experimental data and with the results published so far in numerical studies, which did not take into account the fractal structure of the material and non-locality in time, it was found that the presented results are harmoniously consistent with the existing experimental data. The effect of noisy data on the accuracy of solving the problem was also studied. High levels of noise in the data reduce the accuracy of the solution. But achieving high recovery accuracy required a sufficient number of nodes, which was 60 in each hidden layer. Moreover, increasing the number of nodes in the model was the cause of deterioration in its generalization capabilities when the amount of learning data were limited to about 4000.

To sum up, it should be stated that the use of the developed fPINN for the modeling of heat and moisture transfer in anisotropic material has undoubted advantages and was justified.

## Data Availability

The original contributions presented in the study are included in the article, further inquiries can be directed to the corresponding author.

## Abbreviations

The following abbreviations are used in this manuscript:

Adam	Methods for estimating the adaptive moment
ANN	Artificial neural network
BFGS	Broyden–Fletcher–Goldfarb–Shanno
FDM	Finite difference method
fPINN	Fractal physics-informed neural network
NAdam	Nesterov-accelerated Adam
PINN	Physics-informed neural network
RMSProp	Root Mean Square Propagation
SGD	Stochastic gradient descent
